# Food- and Nutrient-Based Dietary Patterns and Depression in Korean Adults: A Machine Learning Approach Using KNHANES 2016–2021

**DOI:** 10.3390/nu18091333

**Published:** 2026-04-23

**Authors:** Eunje Kim, Youjin Je

**Affiliations:** Department of Food and Nutrition, Kyung Hee University, Seoul 02447, Republic of Korea; bile176@khu.ac.kr

**Keywords:** depression, dietary patterns, machine learning, cross-sectional study, KNHANES, K-means clustering, nutritional epidemiology, mental health

## Abstract

Background/Objectives: Dietary patterns may influence depression, yet findings remain inconsistent, partly due to methodological variation in dietary pattern identification. As data-driven approaches may help reduce subjectivity and improve reproducibility in dietary pattern identification, this study aimed to identify dietary patterns using a machine learning approach and examine their associations with depression among Korean adults. Methods: Using data from 21,321 Korean adults aged 19–64 years from the Korea National Health and Nutrition Examination Survey (2016–2021), we applied K-means clustering to identify dietary patterns based on both food group and nutrient intake. Dietary intake was assessed using a 24 h dietary recall, and depression status was based on physician diagnosis. Results: Three distinct patterns were identified in both food group-based and nutrient-based analyses. In the food group-based analysis, a balanced and diverse dietary pattern (Cluster 3) was associated with lower odds of depression compared with a pattern characterized by overall low food intake (Cluster 1) (OR 0.64; 95% CI, 0.47–0.88; *p* = 0.007) after full adjustment, whereas no significant association was observed for the high processed food pattern (Cluster 2 vs. Cluster 1) (OR 0.73; 95% CI, 0.53–1.01). No significant associations were observed for nutrient-based clusters after full adjustment. Conclusions: Our findings suggest that adherence to balanced and diverse dietary patterns based on whole foods is associated with lower odds of depression. Food group-based clustering approaches may offer more reproducible and interpretable insights than nutrient-based approaches, supporting their potential utility in epidemiological research and public health strategies.

## 1. Introduction

Depression is a leading cause of disability worldwide [1] and imposes substantial societal and economic burdens, underscoring its significance as a major public health challenge [2,3]. Despite the availability of pharmacological and psychological treatments, current approaches to depression management remain insufficient due to limited treatment adherence, high recurrence, and adverse drug reactions [4]. Accordingly, identifying modifiable lifestyle factors with population-level relevance has become an important public health priority. Among these, diet has gained increasing attention as a potentially modifiable factor that may play a role in the prevention of depression [5,6]. Depression has been linked to several lifestyle factors, including physical activity [7], sedentary behavior [8], and smoking status [9].

Previous studies have reported associations between depression and specific nutrients, such as selenium [10], folate [11], vitamin D [12,13], B vitamins [14], and omega-3 fatty acid [15,16,17], as well as specific food groups, including red meat [18], fruits and vegetables [19], and fish [16,17,20]. However, in real-life dietary habits, individuals consume meals composed of various foods and nutrients simultaneously, rather than ingesting single components in isolation, leading to possible interactions between dietary elements [21]. For this reason, dietary pattern analysis has been suggested as a more appropriate and holistic approach, as it accounts for the complexity of total dietary intake and may support the development of more effective nutritional guidelines [22]. In this context, diet can be considered a complex system, where foods are consumed in combination and may exert synergistic or interactive effects. Therefore, examining dietary patterns from both food-based and nutrient-based perspectives may provide a more comprehensive understanding of diet–health relationships.

Despite increasing interest, evidence linking dietary patterns with depression remains inconsistent, in part due to methodological variation in how patterns are defined [23,24,25,26,27,28]. This issue is particularly relevant in the Korean context, where dietary patterns have undergone substantial changes in recent years, including increased consumption of Westernized diets and ultra-processed foods, which may have important implications for mental health outcomes [29,30,31].

This methodological variability is also evident in studies conducted in Korean populations, where healthier dietary patterns have generally been associated with lower levels of depressive symptoms [32,33,34]. However, dietary patterns in these studies have been defined using diverse approaches, including predefined dietary indices such as Mediterranean diet scores, investigator-driven classifications based on selected food groups, or simplified categorizations of dietary behaviors. Such heterogeneity in pattern derivation may limit comparability across studies and may not fully capture the complexity of overall dietary intake. Data-driven approaches such as unsupervised machine learning offer opportunities to derive dietary patterns with reduced subjectivity and improved reproducibility [5,35]. While principal component analysis (PCA) is commonly used to derive dietary patterns, it produces continuous pattern scores that may be less intuitive to interpret at the individual level. In contrast, K-means clustering identifies mutually exclusive groups of individuals with similar dietary characteristics, which may facilitate the interpretation of dietary patterns in population-based studies. Therefore, K-means clustering can serve as a complementary approach for exploring dietary patterns.

This study aimed to identify dietary patterns using a machine learning approach and examine their associations with depression among Korean adults. Using the Korea National Health and Nutrition Examination Survey (KNHANES) 2016–2021 data, we applied K-means clustering, an unsupervised machine learning method, to identify dietary patterns among Korean adults and examined their associations with depression. Emerging evidence suggests that dietary factors may influence depression through several biological pathways, including inflammation, oxidative stress, and the gut–brain axis [36,37,38,39,40,41,42,43]. Diets rich in vegetables, fruits, fish, and whole grains provide bioactive compounds and nutrients that may support mental health through these mechanisms [36,37,44,45]. We hypothesized that a balanced and diverse dietary pattern would be inversely associated with depression. By leveraging machine learning in a nationally representative dataset, this study aims to generate evidence that can inform dietary recommendations and support public health strategies.

## 2. Materials and Methods

### 2.1. Study Population and Data Source

This cross-sectional study was based on data from the 7th (2016–2018) and 8th (2019–2021) KNHANES, a nationwide, cross-sectional survey conducted by the Korea Centers for Disease Control and Prevention Agency and the Korea Ministry of Health and Welfare. The KNHANES is a nationally representative survey of the non-institutionalized civilian population in Korea, using a stratified, multistage, clustered sampling design. It consists of three major components: nutrition survey, health interview survey, and health examination survey [46]. Of the 46,828 participants enrolled in the 7th to 8th KNHANES, 38,068 individuals completed all three components of the survey. We initially excluded 7259 participants aged under 19 years and 8805 participants aged over 64 years. In addition, 820 participants with diabetes or cardiovascular disease and 270 pregnant or lactating women were excluded. 391 individuals with implausible total energy intake (<2092 or >20,920 kJ/day; equivalent to <500 or >5000 kcal/day) and 842 subjects with insufficient information on depression were excluded. After applying all exclusion criteria, a total of 19,681 participants were included in the analysis.

### 2.2. Assessment of Depression

Depression status was identified based on a self-reported physician diagnosis obtained during the health interview component. Participants who answered “yes” to having been diagnosed with depression were classified into the depression group, while those who answered “no” or did not respond were assigned to the non-depression group. For the food intake-based clustering dataset, a total of 709 participants were classified into the depression group, while 16,300 participants were classified into the non-depression group. In the nutrient-based clustering dataset, 826 participants were identified as having depression, and 18,924 participants were classified as not having depression.

### 2.3. Dietary Data and Preprocessing

Dietary intake was assessed through 24 h dietary recall based on the coding scheme of KNHANES. Individual foods were grouped into 23 predefined food categories (whole grains, white rice, noodles, ramen, refined baked goods, processed sandwich meals, cereals, snacks, starchy convenience foods, sugars, legumes, seeds, vegetables, fruits, meat, eggs, fish, dairy, fats and oils, non-alcoholic drinks, alcoholic drinks, condiments, and others). In addition, intake of 24 nutrients (total energy, protein, total fat, saturated fatty acid, monounsaturated fatty acid, polyunsaturated fatty acid, carbohydrate, fiber, sugar, calcium, potassium, magnesium, iron, zinc, vitamin A, vitamin D, vitamin E, vitamin B1, vitamin B2, vitamin C, water, phosphorus, sodium, and cholesterol) was extracted from the same dietary recall data.

All dietary variables were standardized using Z-score transformation (mean = 0, SD = 1). To reduce the influence of extreme values, participants with absolute Z-scores greater than 4 in any of the variables were excluded from both food group-based and nutrient-based analyses.

### 2.4. Clustering Analysis Using Machine Learning

Unsupervised machine learning via K-means clustering was applied separately for food intake variables and nutrient variables to identify distinct dietary patterns. Z-score standardized intake data for both 23 food groups and 24 nutrients were used for clustering, and no additional energy adjustment was applied. After excluding participants with extreme values (|Z| > 4) to minimize the influence of outliers on cluster formation, K-means clustering was performed.

To determine the optimal number of clusters, we evaluated candidate solutions ranging from k = 2 to k = 6 using the cubic clustering criterion (CCC), Pseudo-F statistic, and the proportion of variance explained (R-square). Both the CCC and Pseudo-F statistic were highest at k = 2. However, this solution was considered overly simplistic and insufficient to capture the complexity of dietary patterns. At k = 3, both CCC and Pseudo-F values remained relatively high, while the increase in R-square was most pronounced compared to k = 2 and plateaued thereafter, indicating diminishing returns with additional clusters. Furthermore, the three-cluster solution yielded well-balanced cluster sizes and meaningful, interpretable dietary patterns. Based on these considerations, a three-cluster solution was selected as the optimal structure.

To assess cluster stability, we conducted repeated K-means clustering using 20 random 80% subsamples of the analytic dataset. Across iterations, three distinct dietary patterns were consistently identified. The overall cluster profiles and relative cluster sizes were broadly stable across repetitions, supporting the robustness of the identified clustering structure.

Because standard K-means clustering in SAS does not directly accommodate complex survey weights, the primary clustering analyses were conducted using unweighted standardized dietary variables. In addition, a sensitivity analysis using a survey weight-informed resampling approach was performed to further assess the robustness of the clustering structures.

### 2.5. Covariates

Information on demographic variables (age, sex, household income, and education level), lifestyle factors (smoking status, alcohol consumption, and physical activity), and anthropometric measurements (body mass index (BMI)) were obtained through the health interview component of the KNHANES. Educational level was categorized into three groups: middle school or lower, high school, and college or higher. Household income was divided into quartiles: lower, lower middle, upper middle, and highest. Smoking status was classified as non-smokers, former smokers, and current smokers. The subjects’ alcohol consumption status was classified into three groups (never/rarely, 1–4 times/month, and ≥2 times/week) based on the investigation of the subjects’ drinking experience over the past year. Physical activity was assessed based on the performance of aerobic activity and was classified into three categories: (1) ≥150  min/week of moderate physical activity, (2) ≥75 min/week of vigorous physical activity, or (3) ≥150 min/week of a combination of moderate and vigorous physical activity (1 min of vigorous physical activity was considered equivalent to 2 min of moderate physical activity). We also calculated total energy intake and used it as a continuous variable. Covariates were selected based on prior literature and their established associations with both dietary behaviors and depression, and were included to account for potential confounding in this relationship [47,48].

### 2.6. Statistical Analysis

All statistical analyses were performed using SAS statistical analysis software (version 9.4, SAS Institute, Inc., Cary, NC, USA). We utilized data from the 2016–2021 KNHANES, which employs a nationally representative, multistage, stratified, and clustered probability sampling design. General characteristics of participants were compared across dietary clusters derived from both food group and nutrient-based K-means clustering. Continuous variables were expressed as means and standard errors, and categorical variables were presented as the number and percentage of subjects. Differences across clusters were tested using the PROC SURVEYREG procedure for continuous variables and the PROC SURVEYFREQ procedure with chi-square tests for categorical variables.

To assess the association between dietary patterns and depression, logistic regression analyses were performed using the PROC SURVEYLOGISTIC procedure. Odds ratios (ORs) and 95% confidence intervals (CIs) for depression were calculated for each cluster, using the most balanced and healthy dietary pattern as the reference group. Two models were constructed: Model 1 adjusted for age only, while Model 2 was further adjusted for sex, education level, household income, BMI, smoking status, and physical activity. Statistical significance was defined as a two-sided *p*-value < 0.05.

Additionally, to facilitate interpretation of dietary cluster characteristics, we visualized the mean Z-scores of each dietary variable (food groups and nutrients) across clusters using heatmaps. These visualizations were generated using Python (version 3.12.7, Anaconda Distribution) and Jupyter Notebook.

### 2.7. Model Performance Comparison Across Logistic Regression Models

To compare model performance, we fitted three survey-weighted logistic regression models including: covariates only, food group-based dietary clusters plus covariates, and nutrient-based dietary clusters plus covariates. Model discrimination and fit were assessed using −2 Log Likelihood, Akaike Information Criterion (AIC), Max-rescaled Nagelkerke R^2^, and the area under the receiver operating characteristic curve (AUC). Higher AUC and R^2^ values and lower −2 Log Likelihood and AIC values were interpreted as indicating better model performance.

## 3. Results

### 3.1. General Characteristics and Dietary Patterns Based on Food Group Clustering

**Figure 1:**
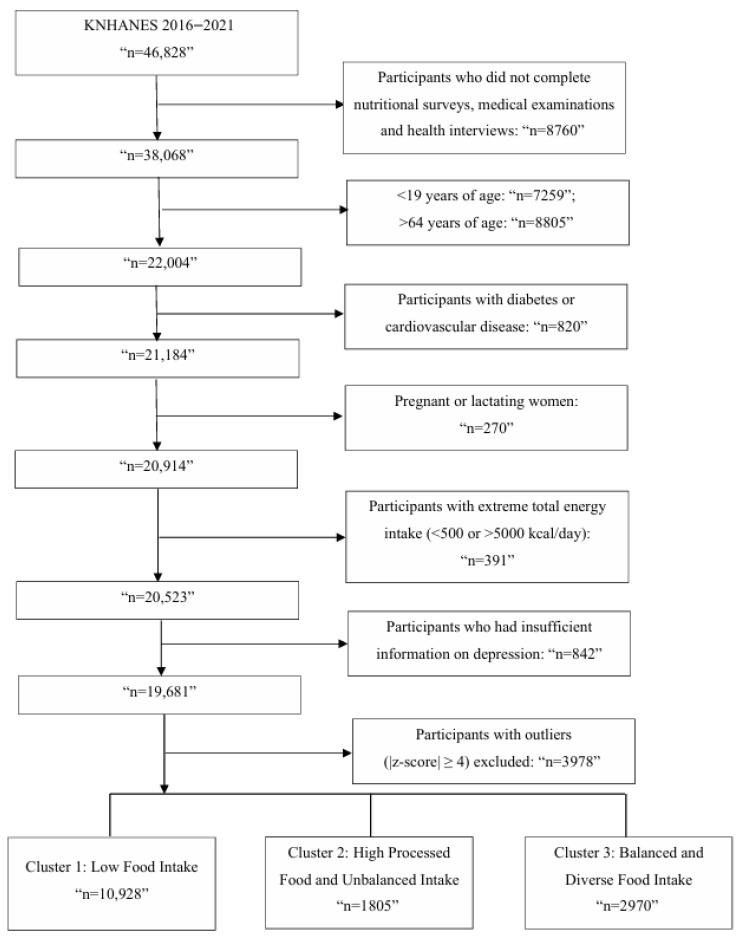
Flow chart of participant selection and food intake-based cluster classification in the KNHANES study.

Among the 19,681 Korean adults included in the analysis, a total of 15,703 individuals were retained in the food group-based clustering dataset after excluding participants with extreme Z-scores (|Z| ≥ 4). As it is shown in Figure 1, three distinct dietary patterns were identified based on intake of 23 food groups by using K-means clustering. The general characteristics of participants according to these clusters are summarized in Table 1.

Cluster 1, which included the largest number of participants (*n* = 10,928), was characterized by uniformly low intake across nearly all food categories. Participants in this group had a mean total energy intake of 1776.7 kcal, indicating a relatively low level of overall dietary intake. The mean age was 42.5 years, and the group had a moderate educational level, with 49.0% having completed college or higher. Smoking status and alcohol consumption were moderate compared with the other clusters, while level of physical activity were the lowest among clusters (47.5%). Overall, this cluster reflected a nutritionally limited diet pattern with relatively favorable smoking and income profiles.

Cluster 2 (*n* = 1805) represented a highly unbalanced diet dominated by processed and convenience foods, consisted of relatively younger individuals with a mean age of 38.6 years, and had the highest proportion of individuals with college or higher education (57.5%). This cluster showed the highest proportion of nonsmokers (65.2%) and a relatively lower proportion of frequent alcohol consumption (21.2% reporting ≥2 times/week). Additionally, this group showed the highest level of physical activity among all clusters (53.7%). This group had the lowest percentage of participants in the lowest income quartile (6.7%). In terms of diet, cluster 2 showed elevated intake of noodles, ramen, refined baked goods, and sugary items, and low intake of vegetables, legumes, and whole grains.

**Figure 2:**
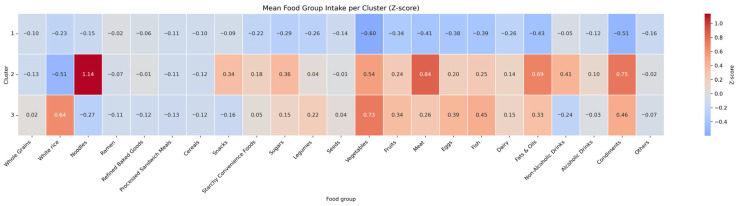
Heatmap of mean food group intake per cluster based on Z-score normalization.

Cluster 3 (*n* = 2970) represented a well-balanced and diverse intake of food groups, and the participants in this cluster were the oldest with a mean age of 44.4 years. Participants of cluster 3 had the highest proportion of individuals whose education level was middle school or less (13.7%), and had fewer individuals in the highest income quartile (37.0%). Notably, this cluster showed the lowest proportions of nonsmokers (52.8%) and the highest proportions of former smokers (23.0%). Additionally, this cluster showed the highest proportion of frequent alcohol consumption (28.0% reporting ≥2 times/week) This group had a higher intake of vegetables, fruits, legumes, fish, and whole grains, as illustrated in the heatmap shown in Figure 2.

### 3.2. Association Between Food Group Clusters and Depression

The associations between dietary patterns derived from food group-based clustering and the odds of depression in Korean adults are presented in Table 2. In the age-adjusted model, both Cluster 2 (OR 0.72; 95% CI, 0.52–0.99) and Cluster 3 (OR 0.56; 95% CI, 0.42–0.77) showed significantly lower odds of depression compared to Cluster 1. In the fully adjusted model, which further accounted for sex, education level, household income, BMI, smoking status, alcohol consumption, physical activity, and total energy intake in addition to age, only the association for Cluster 3 remained statistically significant (OR 0.64; 95% CI, 0.47–0.88). The reduced odds observed in Cluster 2 compared to Cluster 1 (OR 0.73; 95% CI, 0.53 to 1.01) did not reach statistical significance after full adjustment. The overall association among the three clusters remained significant in the multivariable-adjusted model (*p* = 0.007).

### 3.3. General Characteristics and Dietary Patterns Based on Nutrient Clustering

**Figure 3:**
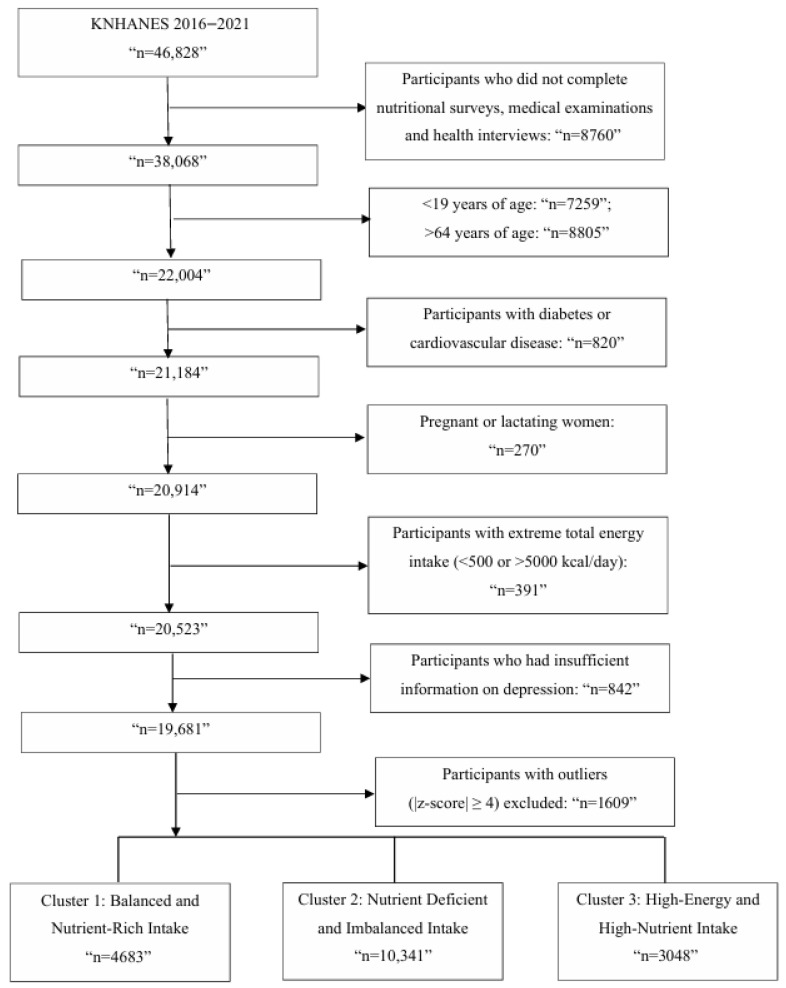
Flow chart of participant selection and nutrient intake-based cluster classification in the KNHANES study.

In the nutrient-based clustering analysis, 18,072 participants remained after exclusion of outliers. Based on Z-score standardized intake of 24 nutrients, three distinct dietary clusters were identified (Figure 3), and the general characteristics of participants across these clusters are presented in Table 3.

**Figure 4:**
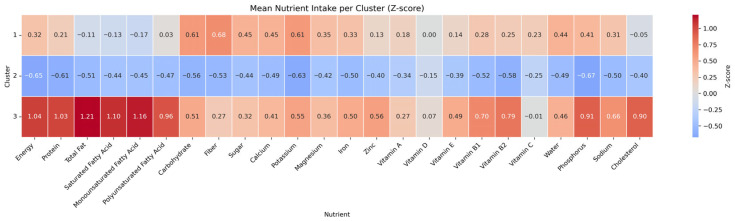
Heatmap of mean nutrient intake per cluster based on Z-score normalization.

The first cluster (*n* = 4683), which represented a balanced and nutrient-rich dietary pattern, included the oldest participants with a mean age of 45.6 years. This group had a relatively moderate proportion of individuals in the highest income quartile (40.4%) and a moderate proportion of college graduates (52.3%). The proportion of nonsmokers (55.9%) and physically active individuals (49.4%) was moderate compared with the other clusters. Subjects in this group showed positive Z-scores across nearly all macro- and micronutrients, including protein, fiber, calcium, iron, and vitamins A, C, and E, indicating an overall high-quality dietary pattern, as visualized in Figure 4.

The second cluster (*n* = 10,341), which was the largest group, exhibited overall nutrient deficiency. Participants in this cluster were younger than participants in cluster 1 with a mean age of 41.6 years, and had the lowest proportion of college graduates (46.7%). They showed the lowest rate of physical activity (47.3%). This cluster showed the highest proportion of nonsmokers (64.5%) and the lowest proportion of frequent alcohol consumption (21.5% reporting ≥2 times/week). In terms of socioeconomic status, this cluster included a higher proportion of individuals in the lowest income quartile (9.5%) and a relatively lower proportion in the highest quartile (35.0%) compared with the other clusters. Additionally, total energy intake was the lowest among the clusters at 1485.9 kcal. This cluster showed negative Z-scores across most nutrients, particularly fiber, calcium, potassium, and essential vitamins.

The third cluster (*n* = 3048) was characterized by the highest intake of energy and nutrients. Participants were the youngest with a mean age of 38.2 years, and had the highest mean BMI (24.2 kg/m^2^). They also had the largest proportion of college graduates (58.5%) and were the most physically active group (52.8%). This cluster also included the highest proportion of current smokers (26.9%), and the highest proportion of frequent alcohol consumption (28.1% reporting ≥2 times/week). While their nutrient intake profile appeared favorable, with high Z-scores for nearly all nutrients, excessive intake of saturated fat, sodium, and cholesterol suggested potential overconsumption rather than optimal balance.

### 3.4. Association Between Nutrient Clusters and Depression

The associations between nutrient-based dietary clusters and the odds of depression are presented in Table 4. In the age-adjusted model, compared with Cluster 1, participants in Cluster 2 showed significantly higher odds of depression (OR 1.22; 95% CI, 1.00–1.49), while Cluster 3 did not show a statistically significant difference (OR 0.81; 95% CI, 0.60 to 1.08). When Cluster 2 was used as the reference group, Cluster 3 showed significantly lower odds of depression (OR 0.66; 95% CI, 0.51–0.85), and Cluster 1 also showed reduced odds (OR 0.82; 95% CI, 0.67–0.996) in the age-adjusted analysis. However, none of these associations remained statistically significant in the multivariable-adjusted model that controlled for sex, education, household income, BMI, smoking status, alcohol consumption, physical activity, and total energy intake in addition to age. The multivariable-adjusted odds ratios were 0.88 (95% CI: 0.68–1.15) for Cluster 2 and 0.95 (95% CI: 0.70–1.30) for Cluster 3, when compared to Cluster 1. The overall association across clusters was no longer statistically significant in the multivariable-adjusted model (*p* = 0.635).

### 3.5. Predictive Performance Comparison of the Three Logistic Regression Models

Table 5 presents the predictive performance of the three logistic regression models. Model B, which included food-based dietary clusters, showed slightly better model fit, as indicated by lower −2 Log Likelihood and AIC values and marginally higher AUC and Max-rescaled R^2^. Although Model C with nutrient-based clusters showed a slight improvement over Model A, the magnitude of improvement was modest.

## 4. Discussion

In this study, we utilized an unsupervised machine learning approach, K-means clustering, to derive dietary patterns based on food group and nutrient intake and investigated their associations with depression using nationally representative data from the Korean population. Traditional dietary pattern analyses, such as factor analysis or predefined diet scores often rely on subjective decisions about food grouping, number of components, or cutoff thresholds, which may lead to inconsistent findings across studies. In contrast, K-means clustering offers a data-driven method for identifying naturally occurring patterns within the population. This method improves reproducibility and minimizes subjectivity in dietary pattern derivation by relying on algorithmic clustering rather than investigator-defined criteria. Furthermore, applying the clustering method separately to both food group and nutrient group enabled us to explore not only nutrient adequacy but also the broader dietary structure and sources of nutrient intake, providing a more comprehensive view of diet–depression associations.

The food group-based clustering provided distinct and interpretable dietary patterns. Cluster 3, a well-balanced and diverse food intake group, was characterized by higher intake of vegetables, fruits, legumes, fish, and whole grains showed the most favorable inverse association with depression. In contrast, no significant association was observed between nutrient-based dietary patterns and depression in fully adjusted models. This attenuation after full adjustment suggests that the apparent associations in nutrient-based models may be partly explained by confounding factors such as lifestyle and socioeconomic variables. These findings highlight the importance of considering whole dietary structures rather than individual nutrients when examining diet and depression relationships, particularly in the context of population-based prevention. Moreover, they may provide a scientific basis for integrating dietary quality considerations into dietary guidelines, community-based nutrition programs, and other public health strategies aimed at mental health promotion.

In addition to the association analyses, model performance comparisons further supported the relevance of food-based dietary clusters in depression research. Models incorporating food-based clusters showed better overall discrimination than the covariate-only model, whereas nutrient-based clusters provided only modest improvements. These findings suggest that dietary information defined at the food-group level provides greater incremental predictive value for depression than information derived from the intake of individual nutrients. This may reflect the fact that food combinations capture the synergistic and interactive nature of diet more effectively than nutrient quantities considered in isolation. In addition, as clustering was based on standardized absolute intake variables rather than energy-adjusted measures, the identified dietary clusters may also capture differences in overall food quantity in addition to dietary quality and composition.

Several biological mechanisms may explain the inverse association between the food group-based Cluster 3 dietary pattern and depression. This dietary pattern was marked by a high intake of vegetables, fruits, legumes, fish, and whole grains—foods that are rich in a variety of nutrients known to support mental health. Fruits and vegetables, which were consumed in greater amounts in Cluster 3, provide antioxidants, dietary fiber, and essential micronutrients that may contribute to lower odds of depression through mechanisms such as reducing oxidative stress and supporting neurotransmitter function [36,37]. Fish, another defining feature of Cluster 3, is a major source of omega-3 polyunsaturated fatty acids, which have been shown to possess anti-inflammatory, neuroprotective effects, and antioxidant properties by reducing oxidative stress [38,39,44,45]. In addition, whole grains may contribute to mental health through pathways such as modulation of the gut–brain axis and regulation of circadian rhythms [40,41,42,43].

While the food group-based clusters revealed meaningful associations with depression, the nutrient-based patterns did not show significant associations in the fully adjusted models. One possible explanation for this discrepancy lies in the inherent limitations of nutrient-based approaches. While nutrient data provide quantitative estimates of individual nutrient intake, they do not fully reflect the complexity and synergy of whole food consumption. Nutrients are not consumed in isolation but rather as part of complex food matrices, where the interaction between various components may play a critical role in influencing mental health outcomes [21]. For instance, the source of a given nutrient, such as whether it is derived from plant-based or animal-based foods, can modulate its bioavailability and physiological effects [49,50,51]. Additionally, foods contain non-nutritive compounds such as phytochemicals, polyphenols, and other bioactive substances that are not accounted for in nutrient-only analyses but may contribute to mental health benefits [52,53].

This study has several strengths. First, we utilized a large, nationally representative sample from KNHANES data, enhancing the generalizability of the findings to the Korean adult population. Second, by employing K-means clustering, an unsupervised machine learning technique, we were able to derive data-driven dietary patterns with minimal subjectivity, improving reproducibility compared to traditional methods. Third, clustering was performed using standardized absolute intake variables, enabling the derived dietary patterns to reflect real-world dietary consumption behaviors by capturing both overall food quantity and dietary composition. Fourth, the dual approach of separately analyzing food group-based and nutrient-based patterns also enabled a more refined understanding of dietary behaviors and their associations with depression. Finally, various covariate adjustments, including sociodemographic and lifestyle factors, helped to minimize potential confounding.

In spite of several strengths, this study has several limitations. First, as KNHANES is a cross-sectional study, causal relationships cannot be inferred, and the observed associations should be interpreted with caution. Reverse causation cannot be ruled out, as depression may influence dietary behaviors. Individuals with depression may adopt less healthy dietary patterns due to reduced motivation, changes in appetite, or emotional eating behaviors. Second, dietary intake was based on a single 24 h dietary recall, which may not accurately reflect habitual dietary patterns and is vulnerable to measurement error. Third, depression was defined based on self-reported physician diagnosis, which may be subject to recall bias and does not capture the severity of depressive symptoms. Fourth, K-means clustering assumes spherical clusters and equal variance, which may not fully capture the complex and heterogeneous structure of dietary data. In addition, although cluster stability was assessed, formal external validation of the clustering structure was not performed. Fifth, survey weights were not incorporated in the primary clustering step due to methodological constraints. Sixth, despite adjusting for multiple covariates, residual confounding cannot be completely ruled out, particularly due to the lack of information on factors such as medication use, comorbidities, and psychiatric history. Healthier dietary patterns may also reflect broader aspects of socioeconomic status and overall well-being, which could partly influence the observed associations. Seventh, selection bias may have occurred due to the exclusion of participants with missing data or pre-existing conditions, which may limit the representativeness of the study population. Eighth, as this study was conducted in a Korean population, the generalizability of the findings to other populations may be limited. Finally, potential sex-specific differences in the association between dietary patterns and depression were not examined and warrant further investigation.

Future studies should aim to address the limitations of the current study. Longitudinal and prospective cohort studies are needed to clarify the temporal and causal relationships between dietary patterns and depression, while intervention studies are required to determine whether dietary modifications can reduce depression risk. In addition, repeated dietary assessments and the use of standardized diagnostic tools may improve measurement accuracy. From a methodological perspective, the application of advanced machine learning approaches and external validation of dietary patterns are warranted. Further research is also needed to explore underlying biological mechanisms and to extend findings to diverse populations.

## 5. Conclusions

In conclusion, dietary patterns characterized by a balanced and diverse intake of whole foods, including vegetables, fruits, legumes, fish, and whole grains, were associated with a lower odd of depression among Korean adults. These findings highlight the importance of overall dietary quality and diversity, rather than isolated nutrients, as modifiable factors in mental health. From a public health perspective, promoting balanced dietary patterns may also contribute to reducing the burden of depression at the population level. However, longitudinal and interventional studies are needed to confirm these associations and to further clarify the role of dietary improvements in depression prevention and mental health promotion.

## Figures and Tables

**Table 1:** General characteristics of the study subjects according to food intake cluster phenotypes among Korean adults aged 19–64 years.

	Food Intake Phenotypes			
	Cluster 1 ^a^	Cluster 2 ^b^	Cluster 3 ^c^	
Variables	N (%) or Mean (SE)			*p*
Number of subjects	10,928	1805	2970	
Age (years)	42.5 (0.2)	38.6 (0.4)	44.4 (0.3)	<0.001
BMI (kg/m^2^)	23.9 (0.05)	23.4 (0.1)	23.9 (0.1)	<0.001
Education				
Less than or equal to middle school	1574 (10.7)	139 (6.0)	517 (13.7)	<0.001
High school	4283 (40.3)	629 (36.5)	1096 (38.2)	
Greater than or equal to college	5057 (49.0)	1035 (57.5)	1353 (48.2)	
Household income				
Lowest	1054 (8.5)	107 (6.7)	241 (7.8)	0.018
Lower middle	2550 (22.3)	367 (20.0)	683 (21.8)	
Upper middle	3394 (31.6)	555 (30.6)	963 (33.3)	
Highest	3898 (37.6)	776 (42.8)	1079 (37.0)	
Smoking				
Nonsmoker	6988 (59.6)	1266 (65.2)	1739 (52.8)	<0.001
Former smoker	2044 (20.8)	264 (16.4)	617 (23.0)	
Current smoker	1884 (19.6)	275 (18.3)	611 (24.2)	
Alcohol consumption				
Never/rarely	4754 (40.4)	724 (35.8)	1108 (34.8)	<0.001
1-4 Times/month	3936 (38.2)	727 (43.0)	1073 (37.2)	
≥2 Times/week	2228 (21.4)	354 (21.2)	787 (28.0)	
Physical activity ^d^				
No	5942 (52.5)	902 (46.3)	1599 (51.7)	<0.001
Yes	4965 (47.5)	899 (53.7)	1367 (48.3)	
Total energy intake (kcal) ^e^	1776.7 (8.8)	2043.1 (16.3)	2069.9 (13.6)	<0.001

All values are means ± standard errors (SE) or N (%). BMI, body mass index. *p*-values represent differences in means or proportions based on ANOVA or chi-square tests. ^a^ Cluster 1: Low food intake; ^b^ Cluster 2: High processed food and unbalanced intake; ^c^ Cluster 3: Balanced and diverse food intake. ^d^ Physical activity was defined as ≥150 min/week of moderate physical activity, ≥75 min/week of vigorous physical activity, or ≥150 min/week of a combination of moderate and vigorous physical activity (1 min of vigorous physical activity was considered 2 min of moderate physical activity). ^e^ Adjusted for age (continuous), body mass index (continuous), education (less than or equal to middle school, high school, or greater than or equal to college), household income (lowest, lower middle, upper middle, or highest), smoking (nonsmoker, former smoker, or current smoker), alcohol consumption (never/rarely, 1–4/month, or ≥2/week), and physical activity (yes or no).

**Table 2:** Odds ratios for depression according to food intake cluster phenotypes among Korean adults aged 19–64 years.

	Food Intake Phenotypes		
	Cluster 1 ^a^	Cluster 2 ^b^	Cluster 3 ^c^
No. of cases/subjects	510/10705	41/1379	158/4925
	OR (95% CI)	OR (95% CI)	OR (95% CI)
Cluster 1 as reference			
Age-adjusted OR (95% Cl)	1.0 (ref.)	0.72 (0.52–0.99)	0.56 (0.42–0.77)
Multivariable-adjusted OR (95% CI) ^d^	1.0 (ref.)	0.73 (0.53–1.01)	0.64 (0.47–0.88)
Cluster 2 as reference			
Age-adjusted OR (95% Cl)	1.40 (1.02–1.93)	1.0 (ref.)	0.79 (0.52–1.20)
Multivariable-adjusted OR (95% CI) ^d^	1.37 (0.99–1.90)	1.0 (ref.)	0.87 (0.58–1.33)
Cluster 3 as reference			
Age-adjusted OR (95% Cl)	1.78 (1.31–2.41)	1.27 (0.84–1.93)	1.0 (ref.)
Multivariable-adjusted OR (95% CI) ^d^	1.57 (1.14–2.15)	1.14 (0.75–1.74)	1.0 (ref.)

*p*-values are based on likelihood ratio tests comparing logistic regression models with and without the cluster variable to assess the overall association. Age-adjusted model: *p* = 0.001; Multivariable-adjusted model: *p* = 0.024. ^a^ Cluster 1: Low food intake; ^b^ Cluster 2: High processed food and unbalanced intake; ^c^ Cluster 3: Balanced and diverse food intake. ^d^ Adjusted for age (continuous), sex, education (less than or equal to middle school, high school, or greater than or equal to college), household income (lowest, lower middle, upper middle, or highest), smoking (nonsmoker, former smoker, or current smoker), alcohol consumption (never/rarely, 1–4/month, or ≥2/week), physical activity (yes or no), and total energy intake (continuous). OR, odds ratio; Cl, confidence interval; ref., reference category.

**Table 3:** General characteristics of the study subjects according to nutrient intake cluster phenotypes among Korean adults aged 19–64 years.

	Nutrient Intake Phenotypes			
	Cluster 1 ^a^	Cluster 2 ^b^	Cluster 3 ^c^	
Variables	N (%) or Mean (SE)			*p*
Number of subjects	4683	10,341	3048	
Age (years)	45.6 (0.2)	41.6 (0.2)	38.2 (0.2)	<0.001
BMI (kg/m^2^)	23.8 (0.1)	23.8 (0.05)	24.2 (0.1)	<0.001
Education				
Less than or equal to middle school	648 (10.7)	1721 (12.7)	163 (4.2)	<0.001
High school	1698 (37.0)	4016 (40.6)	1102 (37.3)	
Greater than or equal to college	2332 (52.3)	4589 (46.7)	1780 (58.5)	
Household income				
Lowest	350 (7.3)	1073 (9.5)	187 (6.1)	<0.001
Lower middle	989 (20.4)	2537 (23.7)	627 (19.4)	
Upper middle	1482 (31.8)	3218 (31.9)	958 (31.7)	
Highest	1855 (40.4)	3484 (35.0)	1273 (42.8)	
Smoking				
Nonsmoker	2801 (55.9)	7117 (64.5)	1497 (45.3)	<0.001
Former smoker	1045 (23.9)	1538 (16.5)	812 (27.8)	
Current smoker	837 (20.2)	1669 (19.0)	738 (26.9)	
Alcohol consumption				
Never/rarely	2081 (41.3)	4536 (41.0)	950 (29.2)	<0.001
1–4 Times/month	1624 (36.6)	3693 (37.5)	1249 (42.7)	
≥2 Times/week	978 (22.0)	2098 (21.5)	848 (28.1)	
Physical activity ^d^				
No	2440 (50.6)	5683 (52.7)	1493 (47.2)	<0.005
Yes	2237 (49.4)	4635 (47.3)	1550 (52.8)	
Total energy intake (kcal) ^e^	2213.7 (7.9)	1485.9 (5.2)	2734.9 (12.2)	<0.001

All values are means ± standard errors (SE) or N (%). BMI, body mass index. *p*-values represent differences in means or proportions based on ANOVA or chi-square tests. ^a^ Cluster 1: Balanced and nutrient-rich intake; ^b^ Cluster 2: Nutrient deficient and imbalanced intake; ^c^ Cluster 3: High-energy and high-nutrient intake. ^d^ Physical activity was defined as ≥150 min/week of moderate physical activity, ≥75 min/week of vigorous physical activity, or ≥150 min/week of a combination of moderate and vigorous physical activity (1 min of vigorous physical activity was considered 2 min of moderate physical activity). ^e^ Adjusted for age (continuous), body mass index (continuous), education (less than or equal to middle school, high school, or greater than or equal to college), household income (lowest, lower middle, upper middle, or highest), smoking (nonsmoker, former smoker, or current smoker), alcohol consumption (never/rarely, 1–4/month, or ≥2/week), and physical activity (yes or no).

**Table 4:** Odds ratios for depression according to nutrient intake cluster phenotypes among Korean adults aged 19–64 years.

	Nutrient Intake Phenotypes		
	Cluster 1 ^a^	Cluster 2 ^b^	Cluster 3 ^c^
No. of cases/subjects	195/5157	548/11,932	83/2661
	OR (95% CI)	OR (95% CI)	OR (95% CI)
Cluster 1 as reference			
Age-adjusted OR (95% Cl)	1.0 (ref.)	1.22 (1.00–1.49)	0.81 (0.60–1.08)
Multivariable-adjusted OR (95% CI) ^d^	1.0 (ref.)	0.88 (0.68–1.15)	0.95 (0.70–1.30)
Cluster 2 as reference			
Age-adjusted OR (95% Cl)	0.82 (0.67–0.996)	1.0 (ref.)	0.66 (0.51–0.85)
Multivariable-adjusted OR (95% CI) ^d^	1.13 (0.87–1.47)	1.0 (ref.)	1.08 (0.74–1.57)
Cluster 3 as reference			
Age-adjusted OR (95% Cl)	1.52 (1.18–1.96)	1.24 (0.93–1.66)	1.0 (ref.)
Multivariable-adjusted OR (95% CI) ^d^	1.05 (0.77–1.43)	0.93 (0.64–1.35)	1.0 (ref.)

*p*-values are based on likelihood ratio tests comparing logistic regression models with and without the cluster variable to assess the overall association. Age-adjusted model: *p* = 0.008; Multivariable-adjusted model: *p* = 0.868. ^a^ Cluster 1: Balanced and nutrient-rich intake; ^b^ Cluster 2: Nutrient deficient and imbalanced intake; ^c^ Cluster 3: High-energy and high-nutrient intake. ^d^ Adjusted for age (continuous), sex, education (less than or equal to middle school, high school, or greater than or equal to college), household income (lowest, lower middle, upper middle, or highest), smoking (nonsmoker, former smoker, or current smoker), alcohol consumption (never/rarely, 1–4/month, or ≥2/week), physical activity (yes or no), and total energy intake (continuous). OR, odds ratio; Cl, confidence interval; ref., reference category.

**Table 5:** Model performance comparison across three logistic regression models for depression.

Model	Variables Included	−2 Log Likelihood	AIC	Max-Rescaled R^2^	AUC
Model A	Covariates only	7,620,357.9	7,620,381.9	0.0607	0.685
Model B	Covariates + Food-based clusters	7,607,534.0	7,607,562.0	0.0626	0.689
Model C	Covariates + Nutrient-based clusters	7,617,968.3	7,617,996.3	0.0611	0.685

AIC, Akaike Information Criterion; AUC, area under the ROC curve; R^2^, Max-rescaled Nagelkerke R^2^. Lower −2 Log Likelihood and AIC and higher AUC and R^2^ indicate better model performance.

## Data Availability

The data analyzed in this study are publicly available from the Korea National Health and Nutrition Examination Survey (KNHANES) database (https://knhanes.kdca.go.kr).

## Abbreviations

The following abbreviations are used in this manuscript:

KNHANES	Korea National Health and Nutrition Examination Survey
BM	body mass index
OR	odds ratio
CI	confidence interval
AIC	Akaike Information Criterion
AUC	area under the receiver operating characteristic curve
